# Health Risk Assessment of Dietary Heavy Metals Intake from Fruits and Vegetables Grown in Selected Old Mining Areas—A Case Study: The Banat Area of Southern Carpathians

**DOI:** 10.3390/ijerph17145172

**Published:** 2020-07-17

**Authors:** Dan Nicolae Manea, Anişoara Aurelia Ienciu, Ramona Ştef, Iosefina Laura Şmuleac, Iosif Ion Gergen, Dragos Vasile Nica

**Affiliations:** 1 Banat’s University of Agricultural Sciences and Veterinary Medicine “King Mihai I of Romania” from Timişoara, 119 Calea Aradului Street, 300001 Timişoara, Romania; manea_dn@yahoo.com (D.N.M.); ienciuani@yahoo.com (A.A.I.); chirita_ramona@yahoo.com (R.Ş.); smuleaclaura@yahoo.com (I.L.Ş.); 2 National Research—Development Institute for Machines and Installations Designed to Agriculture and Food Industry, 6 Ion Ionescu de la Brad Blaj, 013813 Bucharest, Romania; 3 Faculty of Pharmacy, Victor Babes University of Medicine and Pharmacy of Timisoara, 300041 Timişoara, Romania

**Keywords:** heavy metals, soil, fruits, vegetables, risk assessment, THQ, TTHQ, FAAS

## Abstract

In this study, we conducted a noncarcinogenic risk assessment of consuming vegetables and fruits grown in two old mining areas from the Banat area of Southern Carpathians (Romania), Moldova Veche (M) and Rusca Montana (R) and in a nonpolluted reference area located near the village of Borlova (Ref). Concentrations of Fe, Mn, Zn, Cu, Ni, Cd and Pb in soils and commonly eaten vegetables and fruits were measured and used for calculating the weighted estimated daily intake of metals (WEDIM), the target hazard quotients (THQ) and the total target hazard quotients (TTHQ) for normal daily consumption in adults. Levels of certain metals in soils and plants from the R area (Pb) and the M area (Cu) were higher than those measured in the Ref area—and often exceeded normal or even alert-threshold levels. TTHQs for the R area (1.60; 6.03) and the M area (1.11; 2.54) were above one for leafy vegetables and root vegetables, respectively, suggesting a major risk of adverse health effects for diets, including these vegetal foodstuffs. Moreover, THQ and TTHQ indicated a higher population health risk for the R area than for the M area, with the Ref area being a safe zone.

## 1. Introduction

Heavy metals, such as iron (Fe), manganese (Mn), zinc (Zn), copper (Cu), nickel (Ni), cadmium (Cd) and lead (Pb) (hereinafter referred to as metals), are high-profile inorganic pollutants that once extracted from the natural environment and accumulated in plant foodstuffs can pose a serious hazard for both human and environmental health [1,2,3,4]. Sludges, solid wastes, fertilizers, waste waters, vehicular and industrial emissions, and many other industrial byproducts are major sources of soil contamination with metals [5,6,7,8,9]. There is compelling evidence for elevated metal uptake in people consuming food crops, vegetables and fruits grown on anthropically contaminated soils, and/or animal products (e.g., meat, milk, eggs) originating from these areas [10,11,12]. These amounts were often reported to be high enough to affect human health at multiple biologic levels [10,13,14], especially in populations from legacy/actual mining areas, large cities and/or the surrounding suburban areas [10,15,16,17,18,19]. Although trace amounts of essential metals, including Fe, Mn, Zn and Ni, play pivotal roles in the metabolism of living organisms [2,20], at high levels they can pose serious threats to both human and animal health [1,3,21]. Importantly, these essential metals have a normal range of oral intake (Fe 8–18 mg/day, Mn 1.82–3 mg/day, Zn 8–11 mg/day, Cu 0.9 mg/day and Ni 0.5 mg/day), with values beyond this range being associated with either deficiency or toxic effects. For example, high intake of Fe and Mn has been shown to cause severe pathologic alterations, e.g., brain iron deposition in Parkinson’s disease. In addition, Cu surplus has been associated with liver damage, whereas Zn has been linked to adverse Cu–nutrient interactions. Moreover, Zn excess is known to reduce immune function and high-density lipoprotein levels [1,2,3,10]. In contrast, non-essential metals, including Cd and Pb, have no biologic roles [22,23] and are toxic to humans even at trace levels. Thus, high intake of Cd and Pb can induce harmful effects, including oxidative stress, skeletal damages, neurological disorders, immune system damage and even cancer [24], with pregnant women and young children being particularly sensitive to such exposure events [4,25]. As a result, it is of utmost importance for human health to determine the levels of toxic metals precisely and accurately in different foodstuffs [26].

Within the Central and Eastern Europe, the Carpathian Mountains (Carpathians) have served as important sources of ferrous and non-ferrous metals since antiquity [27]. Romania includes half of the Carpathians’ range and has numerous sites with a long history of metal mining and soil contamination and pollution with metals originating from such activities has been frequently reported in these areas [28,29,30,31,32]. There is, however, surprisingly little information about the hazardous effects of metal-contaminated sites on local inhabitants [10]. It is hence necessary to identify the potential of these contamination sources to introduce risk agents into the environment, to estimate the amount of risk agents coming into contact with the human environment, and to quantify the potential human health consequences [33,34,35]. In this context, the main aim of this study was to estimate and understand the potential health risks associated with consumption of vegetables and fruits collected from high-profile old mining areas. To this end, we determined via atomic absorption spectrometry (FAAS) [36] the levels of selected metals (Fe, Mn, Zn, Cu, Ni, Cd, Pb) in arable soils from two areas with long-lasting legacy of historic mining activities; as well as in ten vegetables (parsley root and leaves, carrot root and leaves, onion, lettuce, cucumber, green beans, tomatoes and potatoes) and eight fruits (apricots, peaches, sweet cherries, sour cherries, plums, pears, apples and grapes) routinely included in the human diet. The measured values in soils, fruits and vegetables from a nonpolluted area were used as controls. These analytical data sets were used for estimating the daily dietary intake of the selected metals; and for determining the main parameters that describe their potential health effect using the US EPA methodology [26,35].

## 2. Materials and Methods

### 2.1. Sample Localization and Collection

**Figure 1:**
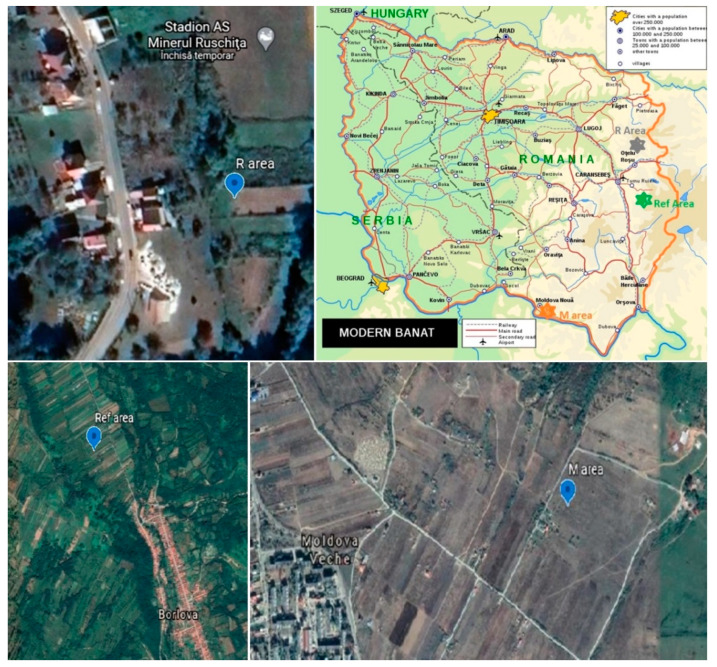
Locations of sampling areas. [37] and Google maps.

The sites investigated were located within the Banat area of Southern Carpathians—an area covering parts from the western and southwestern Romania [37] (Figure 1). More details of the environmental conditions and soil properties have been presented in one of our previous works [10]. Briefly, these areas have a continental Mediterranean climate and a mix of urbic regosols, regosols, mixic entiantrosols (the R area and the M area) and reddish brown soils (the Ref area) [38]. The R area (45°33′40″ latitude N, 22°27′54″ long E; Figure 1) and the M area (44°44′07″ latitude N, 21°37′59″ long E; Figure 1) are well-known for a long history of Pb mining and Cu mining, respectively [27]. In contrast, the Ref area (the reference area, Figure 1) is a relatively nonpolluted area, located in the hilly region of the Caraș-Severin county, near the village of Borlova (latitude = 45°23′01″ latitude N, long = 22°21′12″ long E) and on the right side of Timiş river valley. The surface soils consist mainly of acidic soils with different degree of leaching and erosion process evolved under abundant rainfall (700–900 mm). This site is not only far away from industrial zones, but it is also well-known for its fruit crops obtained using only animal manures as fertilizers [38,39]. This implies that the main source of metals in fruits is related to the geogenic soil metal content. The vegetables and fruits analyzed included carrot (roots and leaves), parsley (roots and leaves), green beans, onion, cabbage, cucumber, tomatoes, potatoes, apricots, sweet cherries, peaches, plums, sour cherries, apples, pears and grapes. All food products were collected from the local vegetable gardens, during the time period May–July 2018 (vegetables), the time period June–August 2018 (fruits) and in September 2018 (potatoes).

### 2.2. Sample Preparation and Analytical Methods

The vegetal foodstuffs were processed in the period 2018–2019 using the protocols routinely used in analytical chemistry for preparing soils and vegetal samples in order to determine their metal content [36,40,41,42]. Briefly, for soils, three samples were collected from the cultivated area (0–20 cm depth) with an agrochemical steel probe and one mixed sample was made from ten single samples. After being transferred to a laboratory environment, the soil samples were air-dried at room temperature, ground and passed through a 2 mm sieve to remove roots, stones and other large particles. For vegetal foods, the analyses were conducted on ten pieces of tomatoes, potatoes, apples, apricots, peaches and pears, whereas for sweet cherries, sour cherries, plums and grapes different amounts (between 100 g and 1000 g) were used. After being washed with deionized water and homogenized, about 100 g of vegetal foodstuff samples were transferred in a quartz capsule, dried at 105 ℃, and, after cooling, homogenized again. Small amounts of dried samples (0.5–1 g) were next treated with aqua regia solution (10 mL 65%-*v/v* HNO_3_ and 30 mL 32%-*v/v* HCl) at 170 ℃ in a digester (Digestion System 6-1007 digester, Tecator). Aqua regia digestion was chosen because it is one of the most common and reliable methods used for analytical determination of metals in soils and vegetal samples [40]. After that, this solution was centrifuged and then brought to 50 mL with double distilled water.

Metal concentrations in filtrates were measured by flame (air–acetylene) atomic absorption spectrometry (VARIAN AA240FS). All analyses were made in triplicate and only the mean values were reported. Results were expressed as milligram per kilogram dry weight (mg/kg). Appropriate stock standard solutions (1000 mg/L) of analytical grade (ICP multielement standard solution IV CertiPUR) and all the other chemicals were purchased from Merck (Darmstadt, Germany). The acetylene was of 99.99% purity (Linde, Romania). All reagents and standard solutions used in our experiments were prepared using spectroscopically pure, double distilled water.

### 2.3. Quality Assurance and Control

It is well-known that the use of atomic absorption spectrometry in air–acetylene flame provides scientists with accurate results and has sufficient sensitivity for detecting metal levels in aqueous solutions resulting from application of aqua regia as a reagent for wet digestion [36,41,42]. In addition, this method is cost-effective, simple and relatively quick [41,42]; this is particularly important for serial measurements, which was the case of our study. The standard calibration curves for different metals were constructed by plotting absorbance against concentration. The coefficients of determination for all metals revealed a good linearity of the obtained data (R^2^ ≥ 0.997), whereas the measured values for relative standard deviations were also excellent (≤9%). The detection limits ranged between 0.01 and 0.015 mg/kg. Blank and duplicate samples were introduced in the series of analyses. The NCS certified reference material-DC 85104a and 85105a (China National Analysis Center for Iron & Steel) were analyzed for quality assurance.

The percent recovery means were: 95% (Fe), 92% (Mn), 102% (Zn), 105% (Cu), 99% (Ni), 91% (Pb) and 105% (Cd). The variation coefficients were below 10%. Metal quantification limits, as determined via the calibration curve, were: 0.15 (Fe), 0.19 (Mn), 0.43 (Zn), 0.13(Cu), 0.14(Ni), 0.01 (Cd) and 0.15 (Pb). The Excel software package (Microsoft Corporation, Redmond, USA) was used for statistical analysis and creation of tables and graphs. The measured values were expressed as means with one standard deviation. Statistical significance was defined at *p* less than 0.05 [40].

### 2.4. Methodology for Health Risk Assessment

The method used here for health risk assessment is based on noncarcinogenic effects, and this risk is expressed abased on target hazard quotients (THQ) [26]. The methodology for estimating this non-numeric parameter is described in detail by the US EPA [43]. This parameter includes not only the intake of metals, but also other relevant parameters, such as exposure frequency, exposure duration, body weight and oral reference dose (RfD). It is calculated by using the following Formula (1):

THQ = (E_F_ × E_D_ × F_IR_ × C) × 10^−3^ / (RfD × W × T_A_) (1)

where E_F_ is the exposure frequency (365 days/year); E_D_ is the exposure duration (75 years), equivalent to the average lifetime [44]; F_IR_ is the food ingestion rate (g/person/day) [45]; RfD is the oral reference dose (mg/kg/day) and is defined as the daily oral exposure to a substance that will not result in any deleterious effect in a lifetime for a given human population; W is the average body weight (64 kg for adults) [46]; and T_A_ is the average exposure time for noncarcinogens (365 days/year × number of exposure years, assuming 75 years in this study). The measured values for each transition metal analyzed here were: 0.700 (Fe), 0.14 (Mn), 0.300 (Zn), 0.04 (Cu), 0.02 (Ni), 0.001 (Cd) and 0.0035 (Pb) [10,19,35]. This variable is a useful tool in environmental risk assessment, e.g., in the EPA’s noncarcinogenic health risk assessment [43].

Assuming that the individual health risks of metals analyzed (THQi) within the same vegetable are cumulative, they can be summed and expressed as a multi-elemental risk hazard index or as total target hazard quotients (TTHQ), which are calculated with the Equation (2) [47,48,49]—see below:

TTHQ = ∑ THQi; i = 1, 2, 3; n = the number of analyzed metals(2)

The concentration-related data, the food ingestion rate, the average body weight, the average lifetime and the RfD-related data were used to calculate the estimated daily intake of metals, the target hazard quotients (THQs) and the multi-elemental risk hazard index TTHQ (also known as the Hazard index, HI) for all transition metals and vegetal foods examined [15,49]. Although both THQ and TTHQ are dimensionless indices, they are rather different in terms of projected outcomes. Thus, the THQ indicates a level of concern, whereas the measured values are additive, but not multiplicative. In contrast, the TTHQ is the sum of various pollutant hazards, in our case metals [19,49]. In terms of interpreting the obtained values, a THQ < 1 indicates that the exposed population is assumed to be safe; 1 < THQ < 4 implies that the exposed population is in a level of concern interval; and THQ > 4 denotes a high level of concern. With respect to TTHQ, TTHQ < 1.0 indicates minimal health impact; TTHQ value > 1.0 denotes potential health risks; and TTHQ > 10.0 suggests serious chronic risk [15].

## 3. Results and Discussion

### 3.1. Metals Concentrations in Soils and Different Vegetal Foodstuffs from Investigated Areas

By investigating metal content in over 15 commonly eaten fruits and vegetables, this study significantly expands previous knowledge on noncarcinogenic risk associated with consumption of vegetal foodstuffs originating from the former mining areas of Banat (Southern Carpathians), which until now was limited to data related to carrot, parsley, onion, green beans, cucumber and cabbage [26]. Soils from these old mining areas are typically rich in complex polymetallic ores with high levels of non-ferrous metals, especially Pb and Cu in the R area and in the M area, respectively. In concordance to our previous studies [10], we found here that Fe tended to be the most abundant metal at these sites, followed by Mn and Zn. The results of soil analyses are given in Table 1.

Metal concentrations in surface soils from the R area decreased in the following order: Pb > Cu > Ni > Cd, whereas in the M area this order was: Cu > Pb > Ni > Cd. All these metals were found, however, at much lower levels in soil samples collected from the Ref area. These data validate our experimental assumption underlying the selection of the Ref area as a reference location in terms of soil metal content.

At all sites, we observed notable differences in metal content when compared to the Romanian Soil Quality regulations related to the admitted metal levels in soils. Manganese was found at high levels at all three sites. At the R site, the measured values were above the normal content (NC) and the alert threshold value (ATV) in Romanian soils that is 1500 mg/kg and 2500 mg/kg, respectively [50]. At the same location, soil Pb has exceeded the intervention threshold value (ITV) (100 mg/kg), whereas the corresponding Zn content was above the ATV level (300 mg/kg). In addition, soil Cd concentration was two-fold higher that the corresponding NC, whereas the Cu level was increased 20-fold. However, at the same location, the amount of Ni in soil fall below the benchmark value for NC (20 mg/kg).

In the M area, Mn and Cu exceeded the ATV level and the ITV level (200 mg/kg), respectively. We also identified above-normal Zn and Pb concentrations at this site, whereas the corresponding amounts of Ni and Cd were below the NC values (20 mg/kg and 1 mg/kg, respectively). These values were close to those measured in the Ref area. Related to the later site, we identified Mn levels above the ATV value and slightly elevated Zn and Cu concentrations (just above the corresponding NC values, i.e., 100 mg/kg and 20 mg/kg, respectively). However, the measured values for Ni, Cd and Pb were below the NC levels (20 mg/kg, 1 mg/kg and 20 mg/kg, respectively) [10]. These values are consistent with geogenic abundance and anthropically induced abundance of metals in Romanian soils [38,39]. Our findings related to the adverse impact of intensive mining and industrial activities on neighboring soils are also in agreement with worldwide data on this topic [16,19,26].

**Figure 2:**
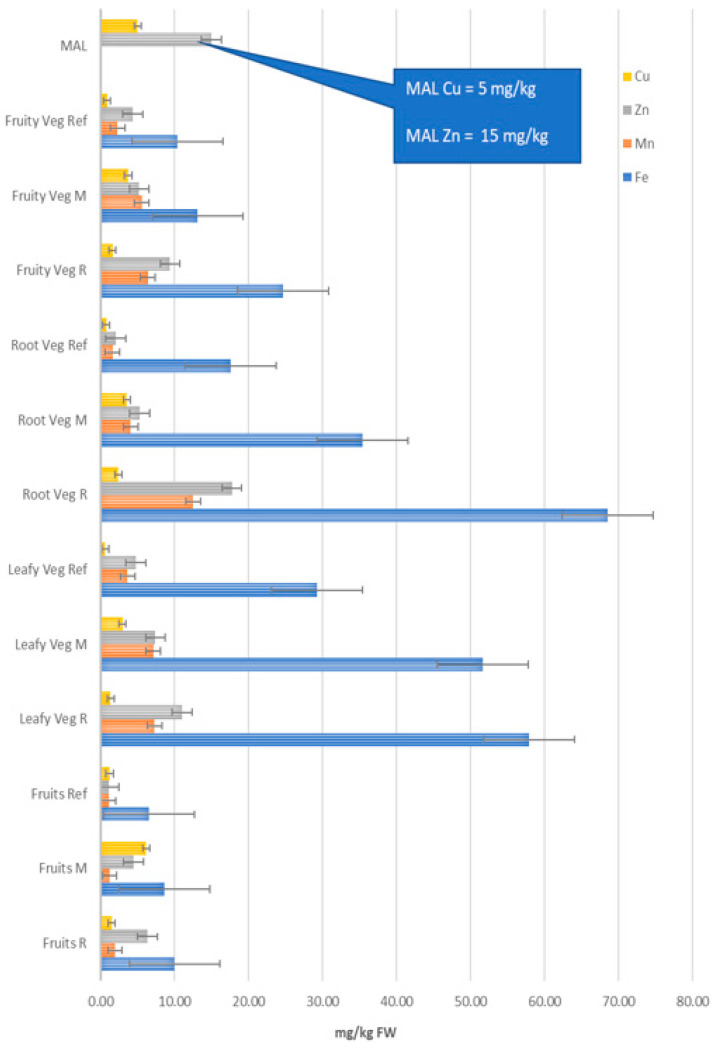
Concentrations of Fe, Mn, Zn and Cu (mg/kg fresh weight (FW)) in different groups of vegetal foodstuffs.

**Figure 3:**
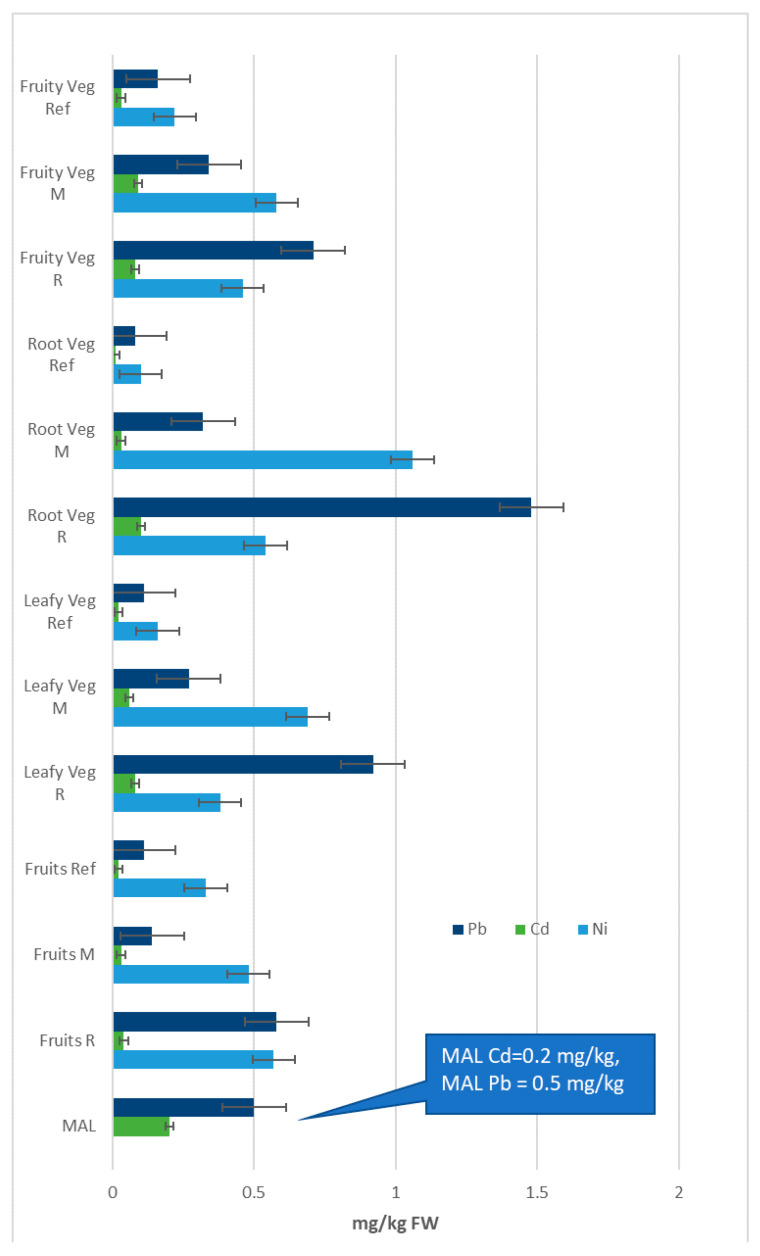
Concentrations of Ni, Cd and Pb (mg/kg FW) in different groups of vegetal foodstuffs.

Metal concentrations in the investigated vegetables are shown in Table 2, Table 3 and Table 4; in the investigated fruits in Table 5. Figure 2 and Figure 3 reveal the levels to which the transition metals analyzed in this study accumulates in different groups of vegetal foodstuffs. The normal amount of Fe in fruits and vegetables is usually relatively high, more precisely between 10 mg/kg and 60 mg/kg fresh weight (FW). In fact, plant foodstuffs serve as the main source of Fe for human nutrition [51,52]. Here, iron concentrations measured here in vegetables and fruits were largely within the aforementioned range, to wit: from 1.6 mg/kg FW up to 222 mg/kg FW in vegetables; and from 5.6 mg/kg FW up to 12 mg/kg FW in fruits, respectively. This essential metal occurred at the highest levels in leafy vegetables and in root vegetables, especially in the R area and the M area. This is likely to be related to the elevated Fe content of soils from these two locations. Although no national or international limits are set for the concentration of Fe in food, chronic intake of 50–100 mg Fe/day can lead to cirrhosis and diabetes [52]. As a result, there are premises that long-term consumption of iron-rich vegetables from these areas (e.g., parsley, carrot, green beans) may negatively affect human health.

In terms of concentration, zinc was the second most abundant metal in this type of foodstuffs. Normal levels of Zn in vegetables and fruits are generally below 10 mg/kg FW, and therefore, these foods are considered to serve as a poor source for this essential metal [51]. Average Zn content determined here in vegetables varied between 0.4 mg/kg FW and 45.8 mg/kg FW. The measured values in fruits were even lower and ranged from 0.97 mg/kg FW up to 8.14 mg/kg FW. In both former mining areas, zinc concentrations exceeding the maximum accepted limit (MAL = 15 mg/kg FW) [53] were frequently found in fruits and vegetables, especially in root vegetables (Figure 2). Similar results were reported for other metal-polluted areas from Romania, such as Baia Mare, Zlatna and Copşa Mică, where the mean Zn content was found to be between 2 mg/kg FW and 9 mg/kg FW [54]. In other parts of the world, higher Zn levels were detected in fruits and vegetables collected at metal-polluted sites: 19.0–42.0 mg/kg FW in Bangladesh [12], 20–44 mg/kg FW in China [47] and 3–54 mg/kg FW in Australia [55], 6–20 mg/kg FW in the Alaverdi’s copper mining complex (Armenia) [16,19], respectively. Comparable concentrations (0.6–6.6 mg/kg FW) were reported for the same vegetal foodstuffs when sampled from Kosovo [56].

Fruits and vegetables regularly contain Mn levels below 50 mg/kg FW [56,57]. In our study, Mn concentrations were between 0.5 mg/kg FW and 10.5 mg/kg FW in vegetables, whereas in fruits the measured values were lower, that is 0.25–3.05 mg/kg FW. Similar to Fe, there are no national or international limits established for Mn in these kinds of foodstuffs. As a result, it is difficult to draw relevant conclusions about the human and environmental relevance of these values.

Normal Cu content in fresh vegetables and fruits should not exceed 3 mg/kg FW [51], whereas the maximum acceptable limit in Romania is 5 mg/kg FW [53]. Copper levels measured in the present study were between 0.23 mg/kg FW and 6.9 mg/kg FW for vegetables and between 0.48 mg/kg FW and 8.61 mg/kg FW for fruits, respectively, with the highest levels being determined in vegetables and fruits from the M area. At this site, the measured values have frequently exceeded the MAL value (Table 2, Table 3, Table 4 and Table 5; Figure 2). Pipoyan has found similar concentrations of Cu in vegetables and fruits cultivated around the Alaverdi’s mining complex (Armenia) [16]. All these findings reflect the history of the M area as one of the largest Cu mining operations in Romania, with major pollution problems and currently under conservation. Importantly, soil Cu was notably above the MAL value, reaching the intervention threshold [10]. Such high Cu concentrations in soils and vegetal foods have been identified in most copper mining areas around the world [10,15,16,55,56].

Average Ni concentrations ranged from 0.17 mg/kg FW up to 0.94 mg/kg FW, with maximum levels being detected in root vegetables from the M area and fruits from the R area. Concentrations close to this range (0.07–2.16 mg/kg FW) have also been found in common vegetables and fruits collected from Bangladesh [12], Armenia [16,19], Kosovo [56], Turkey [58], Brazil [59] and Ethiopia [60]. Related to Pb, the measured values in analyzed vegetables and fruits varied between 0.08 mg/kg FW and 2.45 mg/kg FW, with the maximum values being above MAL (0.5 mg/kg FW) in most fruits and vegetables sampled from the R area. Similarly, fruits and vegetables originating from other Romanian areas with mining legacy revealed a similar range of Pb content, i.e., 1–3 mg/kg FW [54]. These findings are also consistent with the results of other studies around the world on Pb content in vegetal foodstuffs collected from legacy mining areas [15,16,26,56,60].

Globally speaking, Cd in vegetables and fruits varied between 0.01 mg/kg FW and 0.20 mg/kg FW, with the measured values being generally lower than MAL (0.20 mg/kg FW). Elevated Cd levels were found in the R area for root and leafy vegetables, whereas in fruits the measured values were notably lower. Data from the specialty literature reported both similar and higher values in fruits and vegetables grown in areas with comparable degrees of metal pollution [15,19,26,54,56,60,61].

Regardless of the vegetal foodstuff analyzed, elevated metal concentrations were observed primarily in samples coming from areas with long history of mining activities, that is, the M area and the R area-(Table 2, Table 3, Table 4 and Table 5). The highest metal amounts were detected in carrot roots and parsley roots, followed by parsley leaves, carrot leaves, green beans, apricots, peaches and grapes. Similar trends were reported by most scientists investigating metal accumulation patterns in vegetables and fruits collected from areas with different degrees of mining-related pollution/contamination, with differences in metal levels being related to both pedoclimatic conditions and plant species [16,19,26,56,60].

### 3.2. Concentration of Fe, Mn, Zn, Cu, Ni, Cd and Pb in Different Groups of Vegetables

A normal diet cannot practically be expected to include all vegetal products analyzed here, but rather only some of them. As a result, classification of vegetal foodstuffs into four major groups should allow a quick estimation of metal intake following consumption of normal diets containing different combinations of plant foods. Specifically, these four groups are: root & bulb vegetables—hereinafter referred to as root vegetables (carrot root, parsley root, onion, potatoes), leafy vegetables (carrot leaves, parsley leaves cabbage, lettuce), fruiting vegetables (cucumber, tomatoes, green beans) and fruits. The mean metal concentrations in these vegetable groups and the corresponding ranges of individual metals are shown in Figure 2 and Figure 3, whereas the values in the Supplementary Material S1. Because there were notable differences in terms of order of magnitude of metal concentrations, we have presented the data in two separate graphs, i.e., data for Fe, Mn, Zn and Cu in Figure 2; and data for Ni, Pb and Cd (with much lower values) in Figure 3. In both figures we have highlighted the available MAL values. Similar approaches have been already successfully used for calculating specific parameters related to health risk of metal exposure, such as WEDIM and THQ [16,26,56], which are presented below.

The highest concentrations of Fe, Mn, Zn and Cu (Figure 2) were identified, in root vegetables from the R area and in leafy vegetables from the R area and the M area. In all investigated areas, fruiting vegetables showed lower metal concentrations than the other food groups. We found that the MAL values were exceeded only for Zn in root vegetables from the R area; and for Cu in fruits from the M area, respectively. The distribution of highly toxic metals analyzed here (Ni, Cd, Pb) on the four groups of vegetal foodstuffs and the corresponding MAL values are shown in Figure 3.

Ni, Cd and Pb contents of all groups of vegetables were lower in the Ref area relative to the R area and the M area. The highest concentrations of metals with high toxicity (Ni, Cd, Pb) were encountered in root vegetables and leafy vegetables grown in the R area and the M area. We also found that Cd levels and Pb levels in vegetal foodstuffs were below MAL, excepting the vegetal foods collected from the R area which showed values above this threshold. Such excess levels for Pb have also been identified in fruiting vegetables and leafy vegetables collected from the Mojo area farmlands in central Ethiopia [60].

### 3.3. Health Risk Assessment of Metals via Food Consumption

#### 3.3.1. Metal Contribution of the Foodstuff Groups

Most investigations on food metabolomics to date have calculated the daily metal intake separately per each type of food. In this study, we have assessed this indicator as a weighted value, taking into account the share of each type of vegetal food into daily human consumption [16]. This parameter can be obtained by dividing the annual consumption (obtained from the Romanian official statistical data) by the annual average number of days (365). The weighted estimated daily intakes (WEDIM) of Fe, Mn, Zn, Cu, Ni, Pb and Cd for each vegetal foodstuff were calculated via multiplying the weighted average daily intake (fruits 170 g/day, leafy vegetables 150 g/day, root vegetables 370 g/day and fruiting vegetables 130 g/day) [45] by metal concentrations determined in this study or taken from our previous work [10]. Table 6 gives the WEDIM values for all groups of plant foods and all investigated areas, as well as the individual WEDIMs for each analyzed metal and the sums of these values for each group of vegetal foodstuffs and for each area.

Fe intake was dominant in all foodstuffs, especially in root vegetables from the R area and the M area (Table 6). We observed elevated Fe intake for consumption of leafy vegetables collected from the aforementioned locations (Table 6). However, the UL value for this metal (45 mg/kg) can be exceeded only by consuming simultaneously all four groups of vegetables (47 mg/kg) collected from the R area—event which is unlikely to occur in the case of a normal diet.

Related to Zn and Mn, the main sources for these metals were root vegetables and leafy vegetables. The corresponding UL values are far from being achieved via regular consumption of these foodstuffs, irrespective of investigated area (Table 6). Although the Cu intake was elevated for root vegetables from the M area, the UL value for this metal cannot be reached by consuming the analyzed vegetal products (Table 6). On the other hand, the consumption of these foodstuffs fulfills the human need for copper, i.e., 1–2 mg/day [16]. Ni estimated intakes were high only in root vegetables from the R area and the M area. The mixed consumption of these products can lead to exceeding the UL value for Ni; that is 0.2 mg/kg/day, as proposed by EFSA in 2015 [16].

It is worth noting that in all these cases the measured intakes were generally below or close to the corresponding UL thresholds for Fe, Mn, Zn, Cu and Ni in a normal vegetal diet. Indeed, values higher than the UL were found for Cd intake and for Pb intake. However, these values are most probably related to consumption of vegetable foodstuffs collected from the M area and especially from the R area. Pb intake from root vegetables collected from the R area was also above the UL value for Pb. This should raise serious concerns for human health since this nonessential element can be toxic even at trace levels and is very dangerous for nervous system [16,26,56,60]. Because the measured values for WEDIM for Cd in root vegetables from the R area were very close to the UL value for Cd, the consumption of these vegetal foodstuffs is highly likely to serve as a route for introducing Cd into the human body [16,56,60]. Our values were higher than those observed by Mincic in Kosovo [56], Onyedikachi in Nigeria [9], but close to those reported by Pypoian in Armenia [16], Llobet in Catalonia, Spain [22], Gebeyehu in Ethiopia [60], and Li and Zheng in selected industrial and mining areas from China [15,47]. We also observed that metal intakes via fruits were lower than those determined for vegetables, irrespective of analyzed site. Comparable data were reported in other studies investigating metal intake through fruits [16,26].

#### 3.3.2. Target Hazard Quotients (THQ) and Total Target Hazard Quotients (TTHQ)

**Figure 4:**
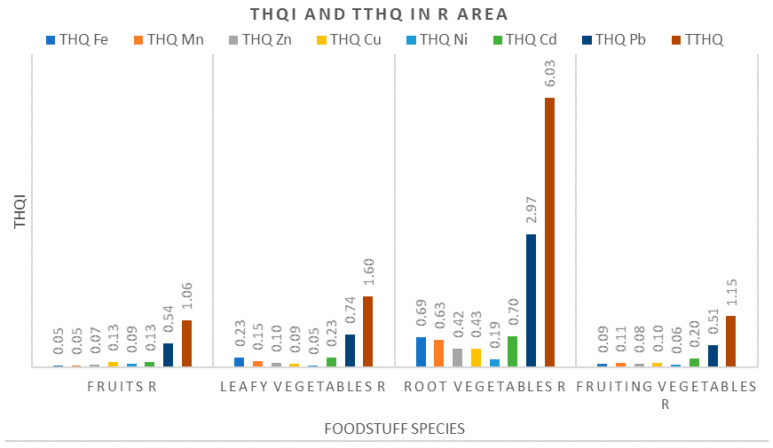
Measured values for individual health risks of metals analyzed (THQi) and total target hazard quotients (TTHQ in the Rusca Montana (R) area.

**Figure 5:**
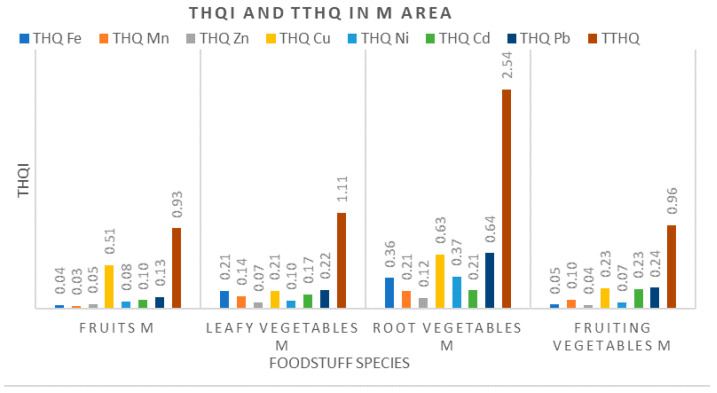
Measured values for THQi and TTHQ in the Moldova Veche (M) area.

**Figure 6:**
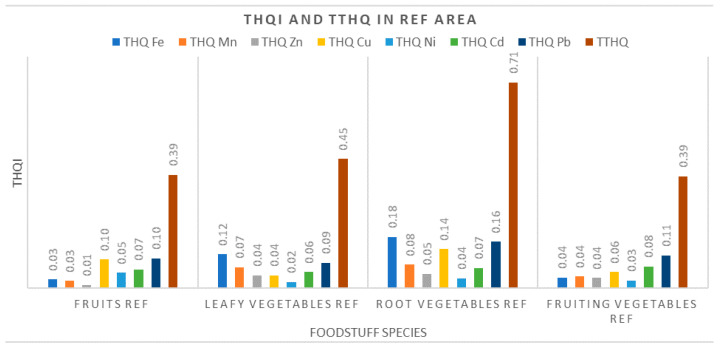
Measured values for THQi and TTHQ in the village of Borlova (Ref) area.

The noncarcinogenic risk to human health associated with consumption of metal-contaminated vegetables and fruits was estimated by using THQ (Equation (1)). The data obtained are shown in Figure 4, Figure 5 and Figure 6. The numeric values are given in the Supplementary Material S1. Assuming the individual additivity of THQs and the linear proportionality of their sum, a total target hazard quotients (TTHQ) was obtained (Equation (2)) for a specific combination of metals in a group of foodstuffs. This index is a measure of the potential risk of adverse health effects from a mixture of chemical constituents identified in a foodstuff [16,19,26,49,56,60,62]. Whether or not the mixture of metals presents an additive risk depends on the targets (tissue, organ or organ system) and their individual mechanisms of action [47,49].

As shown in Figure 4, the highest TTHQs in the R area were identified for the root vegetables, with the measured values far exceeding the critical value of 1. This suggests that, for this site, the potential health risks associated with metal exposure are of high concern. Importantly, most of this risk is related to Pb and Cd intakes, with Pb showing a THQ above 1, and hence serving as a major risk contributor. The same pattern was observed in leafy vegetables. Thus, the TTHQ slightly exceeded the critical value of 1, whereas Pb had the highest contribution to this index. Similarly, the TTHQ values for fruits and fruity vegetables were very close to 1 and again the main contributor to this parameter was Pb. Therefore, one cannot completely exclude that the consumption of these foodstuffs may affect human health, especially the nervous system, the kidneys excretory system and skeletal damage, via the additive effect of these metals [26].

Among the THQ values determined at the R site, only the value of THQ for Pb in root vegetables was above 1. This suggests that consumption of these foods poses the highest health risk to the local inhabitants. However, as described above, by using the TTHQ index we can pertinently extend this warning to all vegetables and fruits grown in the R area.

The measured values for THQ and TTHQ per different groups of vegetal foodstuffs within the M region are shown in Figure 5. In this case, the TTHQs for root vegetables and leafy vegetables were higher than 1, with the main contributor being Cu. A similar pattern of TTHQ, with Cu dominance, was reported by other authors in areas affected by copper mining [16,19,49]. Since the THQs for all metals displayed a value below the critical value, it can be inferred that that the potential health risk associated with the consumption of these plant foods may be related to the combined effect of metals, and especially to Cu. We also observed that the TTHQ indices corresponding to fruits and fruity vegetables were slightly below 1. Therefore, a diet containing these foodstuffs may affect human health via the combined effect of metals cannot be excluded, with the most potentially the harmful effect being related to the increased Cu intake [16]. Although all the measured THQ values in this area stayed below 1, the TTHQ values were above this limit. The later provides evidence that all groups of plants investigated may pose a possible risk to the local inhabitants’ health.

In the Ref area, both the THQ values and the TTHQ values fall below 1 (Figure 6). It was also found that fruits and fruity vegetables showed the lowest values for these variables. This indicates a low metal contamination/pollution at this site, and subsequently a reduced human health risk associated with consumption of vegetal foodstuffs grown here (particularly for root vegetables and leafy vegetables).

Future studies should be extended to cereals and animal foodstuffs to provide a more detailed insight into the health risks associated with consumption of vegetal food stuffs from these areas. It is also important to continue and expand such research to strengthen the scientific basis of local government decisions for appropriate strategic planning aimed at reducing the human health risks in areas exposed to metal pollution/contamination.

## 4. Conclusions

- Metal concentrations in the old mining areas of Moldova Veche (M) and Rusca Montana (R) were above the values measured in the nonpolluted reference area, irrespective of metal analyzed and sample type (soil, vegetal foodstuffs);
- Among metals investigated, Pb and Cu were predominant in the R area and in the M area, respectively. The measured values in soils for these metals at the two former mining sites exceeded the normal limits, reaching the Intervention threshold values for Romania (ITV). These metals were also found at high levels in vegetables and fruit collected from these areas, with root vegetables accumulating the highest levels of metals, followed by leafy vegetables and fruity vegetables;
- Assessment of noncarcinogenic risk for the health of local inhabitants was performed based on the current methodology (US EPA–WHO FAO) via calculating the weighted estimated daily intakes of metals (WEDIM), the target hazard quotients (THQ) and the total target hazard quotients (TTHQ). By using these indices, it was found that consumption of root vegetables (parsley, carrot, onion, potatoes) and leafy vegetables (parsley leaves, cabbage, lettuce) originating from either the M area or the R area is not free of health-related risks;
- Our results indicated that local residents from the R area and the M area can be exposed to hazardous metal levels, especially Pb and Cu, through consumption of vegetables and fruits grown in these areas. The population in the Ref area is not exposed to this risk, both parameters THQ and TTHQ having values well below 1;
- Expansion of present research to other routes of metal exposure, e.g., consumption of other foods (cereals, meat, milk), drinking water and contact with air, is imperative for refining our understanding of populational risk associated with former mining areas.

## Figures and Tables

**Table 1:** Average soil-metal levels (mg/kg) with SD in investigated areas and national limits for these metals [10,50].

Area/Metal Contents	Fe	Mn	Zn	Cu	Ni	Cd	Pb
R Area	56,445.00	5622.33	359.33	49.33	15.00	2.08	136.00
SD	1698.51	375.58	21.23	4.19	0.82	0.18	4.55
M Area	44,548.33	1860.67	203.67	231.00	18.67	0.39	23.00
SD	413.67	53.61	16.13	16.57	2.05	0.07	1.63
Ref Area	32,563.33	2060.33	130.67	24.67	10.00	0.17	14.77
SD	385.81	153.23	11.44	2.49	0.82	0.02	0.71
Normal contents for		900.00	100.00	20.00	20.00	1.00	20.00
Romania (NC)							
Alert threshold values		1500.00	300.00	100.00	75.00	3.00	50.00
for Romania (ATV)							
Intervention threshold		2500.00	600.00	200.00	150.00	5.00	100.00
values for Romania (ITV)							

**Table 2:** Mean (and SD) for metal concentrations in analyzed leafy vegetables (mg/kg FW).

Vegetables	Fe	±SD	Mn	±SD	Zn	±SD	Cu	±SD	Ni	±SD	Cd	±SD	Pb	±SD
MAL					15.00		5.00				0.20		0.50	
					Leafy vegetables									
Carrot leaf R ^#^	31.59 *	5.62	3.13 *	0.64	3.77	0.81	0.4 *	0.08	0.17 *	0.02	0.01 *	0.00	0.20	0.07
Carrot leaf M ^#^	51.45	7.48	5.57	0.40	5.61	0.40	2.12	0.18	0.38	0.09	0.04	0.00	0.12 *	0.00
Carrot leaf Ref ^#^	14.25	1.75	1.38	0.09	1.36	0.36	0.29	0.02	0.11	0.02	0.01	0.00	0.03	0.00
Parsley leaf R ^#^	104.03	26.52	7.32	1.56	9.39 *	1.21	1.77 *	0.11	0.38 *	0.02	0.09	0.01	1.97	0.20
Parsley leaf M ^#^	106.75	13.63	9.72	1.55	10.44 *	2.16	4.79	0.75	1.87	0.19	0.05	0.01	0.50	0.08
Parsley leaf Ref ^#^	73.17	5.51	5.88	0.65	9.13	2.45	1.03	0.27	0.30	0.11	0.03	0.01	0.28	0.07
Cabbage R ^#^	60.11	6.34	10.47	1.46	16.30	3.74	1.36	0.15	0.70	0.11	0.12	0.02	0.90	0.30
Cabbage M ^#^	31.53 *	15.54	9.15	4.24	8.51	4.55	2.77	0.90	0.33	0.18	0.06	0.03	0.25	0.16
Cabbage Ref ^#^	16.06	2.95	3.85	0.80	3.28	0.63	0.45	0.10	0.13	0.03	0.01	0.00	0.05	0.01
Lettuce R ^#^	35.88	4.36	8.33	1.32	14.46	2.77	1.86	0.27	0.28	0.04	0.09	0.01	0.62	0.12
Lettuce M ^#^	16.9 *	2.55	4.12 *	0.60	5.14 *	0.80	2.22	0.40	0.18 *	0.03	0.09	0.01	0.21	0.11
Lettuce Ref ^#^	13.60	1.40	3.46	0.55	5.32	0.68	0.76	0.15	0.10	0.03	0.02	0.00	0.08	0.02

^#^: data from our previous study [10]; * not significant for α < 0.05.

**Table 3:** Mean (and SD) for metal concentrations in analyzed root and bulb vegetables (mg/kg FW).

Vegetables	Fe	±SD	Mn	±SD	Zn	±SD	Cu	±SD	Ni	±SD	Cd	±SD	Pb	±SD
MAL					15.00		5.00				0.20		0.50	
Root and bulb vegetables														
Carrot root R ^#^	29.97	1.57	3.07	0.78	4.93	0.55	1.53	0.09	0.18	0.02	0.08	0.02	2.11	0.31
Carrot root M ^#^	31.90	2.05	2.23 *	0.69	3.17	0.34	1.77	0.11	0.08 *	0.02	0.03	0.00	0.09	0.01
Carrot root Ref ^#^	17.30	0.25	1.43	0.18	2.07	0.27	0.73	0.07	0.04	0.02	0.01	0.00	0.04	0.00
Parsley root R ^#^	221.80	35.12	37.83	4.65	45.83	3.10	5.67	0.97	1.28	0.14	0.20	0.00	2.45	1.96
Parsley root M ^#^	99.00	5.23	8.10	0.65	7.73	0.69	6.87	0.61	3.82	0.64	0.04	0.01	0.66	0.07
Parsley root Ref ^#^	48.77	6.52	2.97	0.36	3.50	0.30	1.23	0.19	0.19	0.03	0.01	0.00	0.08	0.07
Onion R ^#^	15.27	2.23	4.07	0.26	10.90	1.10	0.43 *	0.05	0.21	0.02	0.06	0.01	0.50	0.04
Onion M ^#^	4.67	0.31	1.33	0.11	2.00	0.19	1.37	0.15	0.01	0.00	0.01	0.00	0.13	0.01
Onion Ref ^#^	1.57	0.33	0.33	0.04	0.77	0.07	0.23	0.05	0.03	0.00	0.01	0.00	0.04	0.00
Potato R	12.75	0.27	4.92	0.74	6.02	0.39	2.8 *	0.76	0.63	0.08	0.05	0.01	1.06	0.08
Potato M	10.42	0.77	3.92	0.18	4.14	0.14	7.36	0.89	0.68	0.03	0.04	0.00	0.45	0.08
Potato Ref	8.21	0.66	1.99	0.15	2.35	0.35	1.94	0.15	0.22	0.03	0.02	0.00	0.25	0.04

^#^: data from our previous study; * not significant for α < 0.05.

**Table 4:** Mean (and SD) for metal concentrations in analyzed fruiting vegetables (mg/kg FW).

Vegetables	Fe	±SD	Mn	±SD	Zn	±SD	Cu	±SD	Ni	±SD	Cd	±SD	Pb	±SD
Fruiting vegetables														
Cucumber R ^#^	2.40	0.12	6.73	0.81	8.97	1.12	0.97	0.06	0.28 *	0.03	0.13	0.02	0.72	0.01
Cucumber M ^#^	2.40	0.31	6.00	0.67	1.40 *	0.16	2.40	0.21	0.54	0.06	0.15	0.02	0.37	0.04
Cucumber Ref ^#^	1.70	0.11	0.47	0.05	0.93	1.00	0.47	0.04	0.23	0.03	0.03	0.01	0.16	0.03
Green bean ^#^	58.87	11.29	7.07	0.66	13.23	1.08	1.07	0.15	0.49	0.05	0.06	0.03	0.35	0.07
Green bean M ^#^	26.7 *	5.16	6.77	0.51	10.17 *	1.01	1.43	0.14	0.52	0.05	0.07	0.03	0.19	0.02
Green bean Ref ^#^	21.20	2.19	4.43	0.41	9.67	1.06	0.30	0.08	0.22	0.04	0.03	0.01	0.07	0.02
Tomato R	7.22	0.24	5.10	0.27	9.34	0.58	2.06 *	0.76	0.48	0.06	0.05	0.00	0.85	0.08
Tomato M	6.15	0.82	4.70	0.35	8.33	0.80	4.22	0.43	0.32	0.05	0.05	0.02	0.41	0.02
Tomato Ref	2.87	0.59	1.78	0.20	2.09	0.73	0.89	0.16	0.13	0.04	0.02	0.01	0.18	0.01

^#^: data from our previous study; * not significant for α < 0.05.

**Table 5:** Mean (and SD) for metal concentrations in analyzed fruits (mg/kg FW).

Fruits Spec	Fe	±SD	Mn	±SD	Zn	±SD	Cu	±SD	Ni	±SD	Cd	±SD	Pb	±SD
MAL					15.00		5.00				0.20		0.50	
Apricot R	11.49	1.01	2.53	0.43	8.14	0.16	2.09 *	0.14	0.94	0.12	0.05	0.01	0.74	0.05
Apricot M	9.49 *	0.96	1.81 *	0.16	6.04	0.14	8.02	0.53	0.81	0.06	0.04	0.01	0.15	0.01
Apricot Ref	8.12	0.97	1.59	0.13	1.15	0.16	1.88	0.16	0.45	0.04	0.02	0.01	0.12	0.01
Sw cher R	7.13 *	0.91	2.20	0.45	5.84	0.23	1.27 *	0.09	0.45	0.05	0.04	0.01	0.46	0.04
Sw cher M	6.59 *	1.04	1.14 *	0.15	4.08	0.45	5.22	0.21	0.29	0.04	0.03	0.03	0.15	0.01
Sw cher Ref	5.62	0.51	0.93	0.16	1.17	0.32	1.33	0.16	0.14	0.01	0.02	0.01	0.11	0.02
Peach R	12.27	1.29	2.90 *	0.56	8.09	0.89	1.97	0.14	0.94 *	0.20	0.04	0.01	0.77	0.06
Peach M	10.24 *	1.09	2.26 *	0.25	6.16	0.66	8.61	0.48	0.84 *	0.07	0.04	0.01	0.16	0.01
Peach Ref	9.16	1.07	2.12	0.38	1.07	0.21	1.44	0.13	0.72	0.07	0.02	0.01	0.12	0.01
Plum R	9.20	1.09	1.83	0.38	6.08	0.55	0.88	0.12	0.48	0.07	0.03 *	0.01	0.49	0.02
Plum M	7.16	0.85	0.9 *	0.10	4.39	0.53	4.68	0.28	0.39 *	0.06	0.02 *	0.01	0.15	0.01
Plum Ref	4.35	0.59	0.78	0.06	1.19	0.14	0.48	0.12	0.29	0.04	0.02	0.01	0.10	0.01
S cherry R	10.70	1.16	2.00	0.18	6.84	0.74	2.12	0.14	0.49	0.08	0.05	0.01	0.47	0.06
S cherry M	9.99	0.98	1.01 *	0.10	4.25	0.23	5.26	0.18	0.34	0.05	0.03 *	0.01	0.14	0.01
S cherry Ref	7.35	0.69	0.92	0.10	1.26	0.15	1.45	0.20	0.17	0.02	0.02	0.01	0.10	0.01
Apple R	10.11	1.05	0.65	0.07	4.34	0.28	0.99	0.09	0.36 *	0.06	0.04	0.01	0.56	0.05
Apple M	9.16	1.03	0.46 *	0.04	2.93	0.41	4.91	0.48	0.28 *	0.04	0.02 *	0.01	0.13 *	0.01
Apple Ref	6.51	0.51	0.37	0.08	1.17	0.19	0.82	0.04	0.24	0.04	0.02	0.01	0.11	0.01
Pear R	8.19	0.99	0.55	0.05	4.08	0.08	0.95 *	0.07	0.32 *	0.08	0.03 *	0.01	0.56	0.05
Pear M	6.34	1.07	0.35	0.04	2.79	0.27	4.87	0.44	0.30 *	0.06	0.02 *	0.01	0.11 *	0.01
Pear Ref	3.64	0.57	0.25	0.04	0.97	0.04	0.87	0.04	0.25	0.05	0.02	0.01	0.11	0.01
Grape R	11.15	1.08	3.05	0.22	7.11	0.30	1.99	0.10	0.58	0.08	0.03	0.01	0.61	0.11
Grape M	10.08	1.01	1.88	0.08	4.81	0.33	7.71	0.23	0.55	0.05	0.03	0.01	0.16	0.01
Grape Ref	7.87	0.86	1.88	0.12	0.98	0.11	1.50	0.08	0.35	0.05	0.02	0.01	0.12	0.01

* not significant for α < 0.05.

**Table 6:** The measured values for weighted estimated daily intakes (WEDIM) per all metals, sum of individual WEDIMs for each group of foodstuffs and each area (mg/day/person) and the upper acceptable limit (UL), as compiled from the specialty literature [10,16].

Foodstuff	WEDIM Fe	WEDIM Mn	WEDIM Zn	WEDIM Cu	WEDIM Ni	WEDIM Cd	WEDIM Pb
UL (mg/day/person)	45	11	40 (25)	10	1 (0.2)	0.064	0.24
Fruits R	2.11	0.41	1.33	0.32	0.12	0.01	0.12
Leafy vegetables R	10.42	1.32	1.98	0.24	0.07	0.01	0.17
Root vegetables R	30.85	5.63	7.99	1.09	0.24	0.05	0.67
Fruiting vegetables R	3.95	1.03	1.51	0.26	0.07	0.01	0.11
SUM R area	47.33	8.39	12.80	1.91	0.50	0.08	1.07
Fruit M	1.81	0.26	0.93	1.29	0.10	0.01	0.03
Leafy vegetables M	9.30	1.29	1.34	0.54	0.12	0.01	0.05
Root vegetables M	15.94	1.84	2.39	1.60	0.48	0.01	0.14
Fruiting vegetables M	2.11	0.89	0.84	0.60	0.09	0.01	0.05
SUM M area	29.16	4.27	5.50	4.03	0.79	0.05	0.28
Fruit Ref	1.38	0.23	0.24	0.26	0.07	0.00	0.02
Leafy vegetables Ref	5.27	0.66	0.86	0.11	0.03	0.00	0.02
Root vegetables Ref	7.93	0.73	0.95	0.35	0.05	0.00	0.04
Fruiting vegetables Ref	1.66	0.37	0.69	0.14	0.04	0.00	0.03
SUM Ref area	16.24	1.99	2.73	0.86	0.18	0.02	0.10

## Supplementary Materials

The following are available online at https://www.mdpi.com/1660-4601/17/14/5172/s1, Supplementary Material S1: Metals in groups of Vegs&fruits.
